# PE/PPE mutations in the transmission of *Mycobacterium tuberculosis* in China revealed by whole genome sequencing

**DOI:** 10.1186/s12866-024-03352-y

**Published:** 2024-06-10

**Authors:** Wei-wei Fang, Xiang-long Kong, Jie-yu Yang, Ning-ning Tao, Ya-meng Li, Ting-ting Wang, Ying-Ying Li, Qi-lin Han, Yu-zhen Zhang, Jin-jiang Hu, Huai-chen Li, Yao Liu

**Affiliations:** 1 Department of Respiratory and Critical Care Medicine, Shandong Provincial Hospital, Shandong First Medical University, Jinan, Shandong 250021 PR China; 2 Shandong First Medical University & Shandong Academy of Medical Sciences, Jinan, Shandong People’s Republic of China; 3 Shandong Artificial Intelligence Institute, Qilu University of Technology & Shandong Academy of Sciences, Jinan, Shandong PR China; 4 Shandong University of Traditional Chinese Medicine, Jinan, Shandong PR China

**Keywords:** *Mycobacterium tuberculosis*, Whole genome sequence, PE/PPE, Transmission, China

## Abstract

**Objective:**

This study aims to examine the impact of PE/PPE gene mutations on the transmission of *Mycobacterium tuberculosis* (*M. tuberculosis*) in China.

**Methods:**

We collected the whole genome sequencing (WGS) data of 3202 *M. tuberculosis* isolates in China from 2007 to 2018 and investigated the clustering of strains from different lineages. To evaluate the potential role of PE/PPE gene mutations in the dissemination of the pathogen, we employed homoplastic analysis to detect homoplastic single nucleotide polymorphisms (SNPs) within these gene regions. Subsequently, logistic regression analysis was conducted to analyze the statistical association.

**Results:**

Based on nationwide *M. tuberculosis* WGS data, it has been observed that the majority of the *M. tuberculosis* burden in China is caused by lineage 2 strains, followed by lineage 4. Lineage 2 exhibited a higher number of transmission clusters, totaling 446 clusters, of which 77 were cross-regional clusters. Conversely, there were only 52 transmission clusters in lineage 4, of which 9 were cross-regional clusters. In the analysis of lineage 2 isolates, regression results showed that 4 specific gene mutations, PE4 (position 190,394; *c.46G > A*), PE_PGRS10 (839,194; *c.744 A > G*), PE16 (1,607,005; *c.620T > G*) and PE_PGRS44 (2,921,883; *c.333 C > A*), were significantly associated with the transmission of *M. tuberculosis*. Mutations of PE_PGRS10 (839,334; *c.884 A > G*), PE_PGRS11 (847,613; *c.1455G > C*), PE_PGRS47 (3,054,724; *c.811 A > G*) and PPE66 (4,189,930; *c.303G > C*) exhibited significant associations with the cross-regional clusters. A total of 13 mutation positions showed a positive correlation with clustering size, indicating a positive association. For lineage 4 strains, no mutations were found to enhance transmission, but 2 mutation sites were identified as risk factors for cross-regional clusters. These included PE_PGRS4 (338,100; *c.974 A > G*) and PPE13 (976,897; *c.1307 A > C*).

**Conclusion:**

Our results indicate that some PE/PPE gene mutations can increase the risk of *M. tuberculosis* transmission, which might provide a basis for controlling the spread of tuberculosis.

**Supplementary Information:**

The online version contains supplementary material available at 10.1186/s12866-024-03352-y.

## Background

Tuberculosis (TB) continues to be one of the most prevalent and deadly communicable disease that is a major global health challenge [1]. The Covid-19 pandemic has disrupted TB services which left many TB patients undiagnosed and untreated, leading to an increase in TB deaths and transmission [2, 3]. The evolution and spread of tuberculosis threaten to undermine the success of tuberculosis treatment and control programs [4]. In order to effectively combat TB, it is crucial to have a comprehensive understanding of TB’s transmission mechanisms.

One hallmark of the *Mycobacterium tuberculosis* (*M. tuberculosis*) genome is the presence of the multigenic PE/PPE family of proteins, accounting for about 10% of the coding region of the genome [5]. The standard H37Rv has 99 PE genes and 69 PPE genes, which characterized by conserved N-terminal prolineglutamate (PE) and proline-proline-glutamate (PPE) motifs [6]. Based on the high polymorphism of C-terminal amino acid sequence, the PE family can be divided into PE_PGRS (polymorphic GC-rich sequences) and PE (with no distinctive features) genes, and the PPE family includes PPE_MPTR (major polymorphic tandem repeats), PPE_SVP (with a GxxSVPxxW motif), PPE_PPW(with a PxxPxxW motif) and PPE genes with no distinctive features [5, 7–9]. The unique sequences of PE/PPE proteins might underlie their specific physiological roles during *M. tuberculosis* infection.

Many PE/PPE proteins have been shown to play an important role in antigenicity, immune-modulation and virulence in *M. tuberculosis* [10–12]. For example, PPE68 and PE35 were identified as required for *M. tuberculosis* virulence [13, 14]. Cell necrosis is associated with the spread and virulence of *M. tuberculosis* because it leads to the release and dissemination of the tuberculin pathogen [15]. This function has been reported for PE_PGRS33 [16]and PPE27 [17]. PPE39 [18], PE_PGRS5 [19] and PE_PGRS17 [20] were shown to play roles in host cell interaction and immune regulation. Alternatively, the highly cellular immune response suggests that some PE/PPE proteins may be better diagnostic and vaccine candidates [21].

Some genes were found to contribute to enhanced transmission of *M. tuberculosis*, such as mutation in the ESX-5 type VII secreted protein EsxW [22] and mutation in the lldD2 promoter [23]. The role of PE/PPE genes in transmission, albeit less studied, has shown that mutations in PPE54, for instance, contribute to the enhanced spread of the disease in Malawi [24], highlighting their potential significance in the epidemiological dynamics. Here, we have used whole genome sequence data from 3202 Chinese isolates and detected homoplastic single nucleotide polymorphisms (SNPs) in PE/PPE gene region to assess the impact of these homoplastic SNPs on the spread of *M. tuberculosis*. The result may provide new insights into the impact of the PE/PPE gene mutations on the spread of *M. tuberculosis* in China.

## Method

### Sample collection

The *M. tuberculosis* strains analyzed in this study included two sets of samples (Supplementary Table 1). (1) Newly sequenced whole genome dataset. We included 1,550 culture-confirmed TB samples with drug susceptibility test (DST) results reported to Shandong Tuberculosis Surveillance System during 2013–2017, and the genome sequence data were deposited in the National Center for Biotechnology Information (NCBI) BioProject database (Accession number is PRJNA1002108). (2) Countrywide collection of publicly available clinical isolates of *M. tuberculosis*. We downloaded the WGS data of 1,755 isolates from the European Nucleotide Archive repository. The genomes were sampled from 2007 to 2018, and the geographic distribution covered 30 of the 34 provincial regions of China. Individual patient identifiers were removed before data analysis and reporting. An informed consent waiver and ethical approval were obtained from the Ethics Committee of Shandong Provincial Hospital affiliated to Shandong First Medical University.

### Whole-genome sequencing

The genome of the 1468 Shandong isolates was sequenced using Illumina HiSeq 4000. Quality assessment of all acquired reads was performed with FastQC v.0.11.9 (version 0.11.7), and 1447 samples passed quality control [25]. Low-quality raw reads from paired-end sequencing were discarded. Reads were then aligned to the H37Rv (NC_000962) reference genome using BWA-MEM (version 0.7.17-r1188) [26]. Duplicate reads and clipped alignments were removed with Sam tools markdup (version 1.15) and Samclip (version 0.4.0) [27, 28], and only samples with a coverage rate of 98% or higher and a minimum depth of at least 20 were included. The filtered vcf file was annotated with snpEFF (version 4.3t) to obtain the final sample single nucleotide polymorphisms (SNPs) [29].

### Homoplastic SNPs identifcation

We used snippy-core (version 4.6.0) to obtain SNPs form entire 3202 isolates. To assign lineages, we analyzed *M. tuberculosis* WGS data using TBProfiler (version 4.3.0) [30, 31]. The IQ-TREE (v1.6.12) model “JC + I + G4” used 1000 ultrafast bootstrap replicates and treetime (v0.9.0) to construct and date maximum-likelihood (ML) phylogenetic tree [32]. The occurrence of homoplastic mutations can be attributed to convergent evolution driven by selective pressures. Homoplasy analysis was performed employing the HomoplasyFinder software, in adherence to established protocols and methodologies. The SNPs were considered as homoplastic if they occurred independently within different transmission clades and did not form a monophyletic clade based on the provided phylogenetic tree and nucleotide alignment consistency index [33, 34]. Homoplastic SNPs located in the PE/PPE gene region, with a minor allele frequency (MAF) > 0.005 were included for further analysis.

### Transmissibility analysis

Clusters were defined as strains with a genetic distance of 10 SNPs or fewer, indicating recent transmission [22]. These clusters were categorized as cross-regional or regional, depending on whether they included strains from two or more of China’s seven geographic regions (Northwest, Northern, Northeast, Central, Eastern, Southern, and Southwest China) [35]. Furthermore, transmission clusters were subdivided into small clusters (2 isolates), medium-sized clusters (3–6 isolates), and large clusters (more than 6 isolates) based on the number of isolates within each cluster [36, 37].

### Statistical analysis

To streamline the analysis, homoplastic SNPs with a MAF < 0.005 (approximately 15.

isolates) in the PE/PPE gene region were excluded from the analysis. Between clustered and non-clustered, as well as cross-regional and regional clusters, we employed univariate regression analysis and included sites with *P* values < 0.2 in the subsequent multivariate regression analysis [38]. To analyze the effect of PE/PPE gene mutations on cluster size, we conducted a Spearman’s rank correlation analysis. The statistical analysis was performed using IBM SPSS Statistics (version 26.0). All reported statistical tests were two-sided, and *P* values < 0.05 were considered statistically significant.

## Results

### Sample structure

**Fig. 1:**
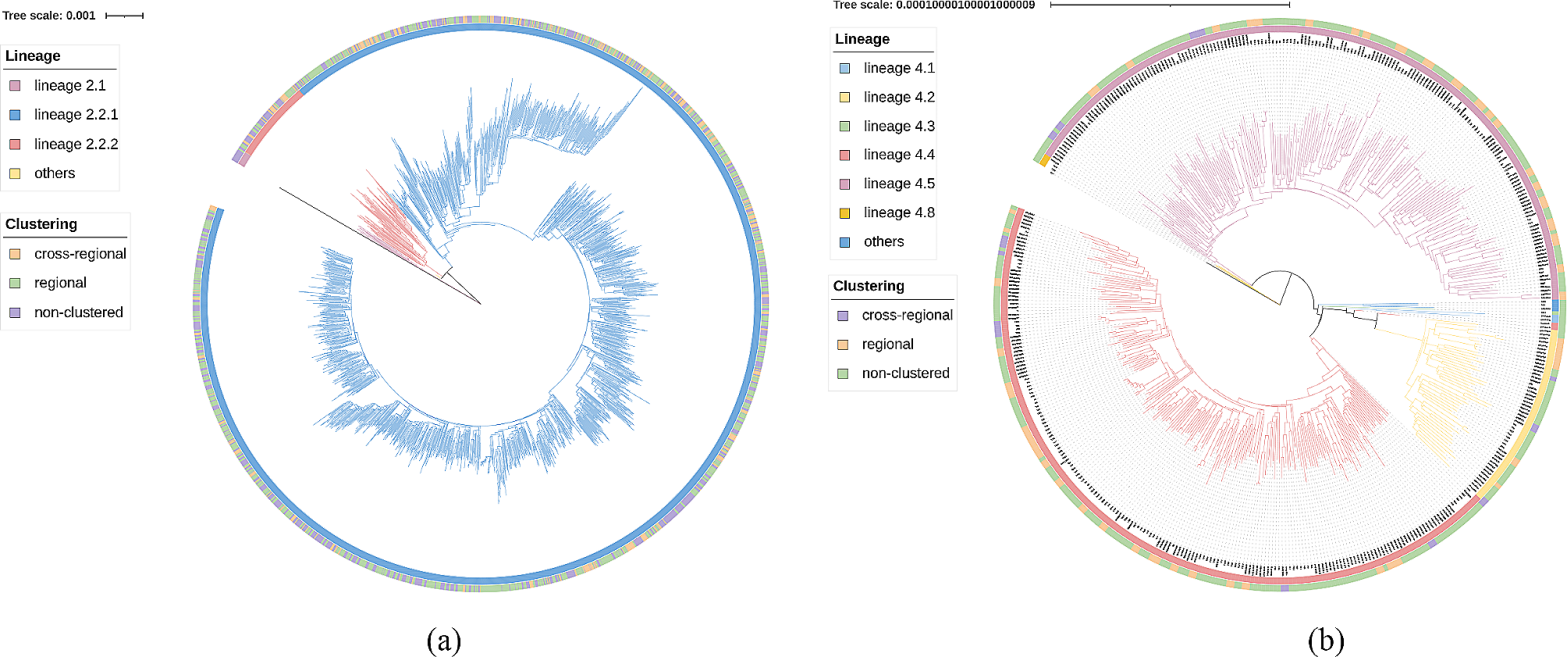
(**a**) Phylogenetic tree of 2745 Chinese *M. tuberculosis* strains in lineage 2. (**b**) Phylogenetic tree of 443 Chinese *M. tuberculosis* strains in lineage 4

Of all domestic 3202 strains, 2745 isolates (85.7%) belonged to lineage 2 (94.4% belonged to sublineage 2.2.1), 443 (13.8%) isolates belonged to lineage 4, only 14 isolates belonged to other lineages (lineage 1 and lineage 3). We constructed maximum likelihood phylogenetic trees for lineage 2 and lineage 4 *M. tuberculosis* isolates, respectively (Fig. 1a and b). The results of strain clustering showed that 1462 strains in lineage 2 were grouped into 446 transmission groups, which were consisted of 2 to 107 isolates. In lineage 4, a total of 52 clusters contained 132 isolates, ranging in size from 2 to 9 isolates (Table 1). It should be noted that lineage 2 strains had a considerably larger proportion of strains in transmission clusters than lineage 4 strains (53.3% vs. 29.8%, *P* < 0.001).

**Table 1:** Lineage and demographic factors associated with transmission clusters (≤ 10 SNP) of *M. tuberculosis* strains in China

Lineage	Clustered No (%)		Non-clustered No (%)	Total (*n* = 3202) No (%)
	Regional	Cross-regional		
1	0 (0.00)	0 (0.00)	4(100.00)	4 (100.00)
2	1080 (39.34)	382 (13.92)	1283 (46.74)	2745 (100.00)
3	2 (20.00)	0 (0.00)	8 (80.00)	10 (100.00)
4	107 (24.16)	25 (5.64)	311 (70.20)	443 (100.00)

### The effect of PE/PPE gene mutations on transmission of L2 strains

A comprehensive analysis revealed 1,141 homoplastic SNPs in lineage 2 strains, as detailed in Supplementary Table 2. After excluding SNPs with a MAF below 0.005, a total of 140 homoplastic SNPs from the PE/PPE gene region were selected for further analysis. Comparing clustered and non-clustered strains, 45 mutation sites with statistical significance (*P <* 0.05) in the univariate regression analysis. To further investigate these associations, the 59 loci with *P* values less than 0.2 in the univariate analysis were included in a multivariate logistic regression analysis. The results indicated that 9 sites were identified as influencing factors (*P* < 0.05), with PE4 (position 190,394; *c.46G > A*; OR, 2.183; 95% CI, 1.025–4.651), PE_PGRS10 (839,194; *c.744 A > G*; OR, 1.668; 95% CI, 1.220–2.280), PE16 (1,607,005; *c.620T > G*; OR, 3.741; 95% CI, 2.039–6.864) and PE_PGRS44 (2,921,883; *c.333 C > A*; OR, 12.664; 95% CI, 1.696–94.357) considered as risk factors for strain clustering (Table 2).

**Table 2:** Analysis of the PE/PPE gene mutations in clustering and non-clustering of lineage 2

**Position**	**Gene**	**SNP**	**Effect**	**MAF**	**OR(95%CI)**	**P-value**
188,800	PE3	*c.40 A > G*	*p.Thr14Ala*	0.928	1.389 (0.254–7.587)	0.704
190,394	PE4	*c.46G > A*	*p.Ala16Thr*	0.009	**2.183 (1.025–4.651)**	**0.043**
333,892	PE_PGRS3	*c.2419 C > G*	*p.Arg807Gly*	0.375	1.277 (0.952–1.712)	0.103
337,820	PE_PGRS4	*c.1254 C > T*	*p.Gly418Gly*	0.085	-	0.998
338,020	PE_PGRS4	*c.1054T > G*	*p.Cys352Gly*	0.059	-	0.998
338,100	PE_PGRS4	*c.974 A > G*	*p.Asn325Ser*	0.072	-	0.998
340,132	PPE3	*c.769G > A*	*p.Glu257Lys*	0.823	-	-
340,372	PPE3	*c.1009T > C*	*p.Ser337Pro*	0.886	0.656 (0.383–1.124)	0.125
340,885	PPE3	*c.1522G > A*	*p.Gly508Ser*	0.011	0.669 (0.334–1.342)	0.258
372,913	PPE6	*c.2799T > G*	*p.Gly933Gly*	0.813	**0.311 (0.116–0.834)**	**0.020**
430,332	PPE8	*c.4348T > C*	*p.Phe1450Leu*	0.013	0.478 (0.046–4.991)	0.537
531,901	PPE10	*c.314 C > A*	*p.Ala105Glu*	0.008	0.653 (0.154–2.759)	0.562
673,564	PE_PGRS7	*c.2353G > A*	*p.Ala785Thr*	0.190	1.251 (0.945–1.656)	0.118
836,087	PE_PGRS9	*c.387 C > T*	*p.Gly129Gly*	0.007	2.273 (0.429–12.045)	0.335
836,426	PE_PGRS9	*c.726 A > C*	*p.Leu242Leu*	0.109	1.412 (0.673–2.962)	0.362
836,454	PE_PGRS9	*c.754 A > G*	*p.Thr252Ala*	0.111	0.53 (0.244–1.151)	0.109
836,538	PE_PGRS9	*c.838 A > G*	*p.Asn280Asp*	0.508	1.099 (0.89–1.358)	0.381
838,990	PE_PGRS10	*c.540 C > G*	*p.Ala180Ala*	0.288	**0.477 (0.356–0.638)**	**<0.001**
839,194	PE_PGRS10	*c.744 A > G*	*p.Thr248Thr*	0.302	**1.668 (1.220–2.280)**	**0.001**
839,684	PE_PGRS10	*c.1234G > A*	*p.Ala412Thr*	0.012	2.199 (0.196–24.727)	0.523
847,613	PE_PGRS11	*c.1455G > C*	*p.Glu485Asp*	0.017	**0.462 (0.271–0.789)**	**0.005**
1,212,432	PE_PGRS21	*c.873 C > A*	*p.Gly291Gly*	0.307	0.863 (0.630–1.182)	0.360
1,218,896	PE_PGRS22	*c.2428G > A*	*p.Gly810Ser*	0.399	0.960 (0.770–1.197)	0.720
1,299,305	PPE17	*c.500 C > T*	*p.Pro167Leu*	0.784	-	0.997
1,561,939	PPE20	*c.171G > C*	*p.Glu57Asp*	0.743	4.322 (0.917–20.359)	0.064
1,572,825	PE_PGRS25	*c.1033G > C*	*p.Ala345Pro*	0.005	-	0.999
1,606,673	PE16	*c.288G > T*	*p.Ala96Ala*	0.800	0.498 (0.13–1.91)	0.309
1,607,005	PE16	*c.620T > G*	*p.Leu207Arg*	0.020	**3.741 (2.039–6.864)**	**<0.001**
1,655,943	PE_PGRS29	*c.779T > A*	*p.Phe260Tyr*	0.025	1.657 (0.519–5.291)	0.394
1,862,424	PE_PGRS30	*c.2959G > A*	*p.Gly987Ser*	0.006	12.414 (0.727-211.884)	0.082
1,931,718	PPE22	*c.937 C > G*	*p.Leu313Va*	0.794	0.338 (0.109–1.05)	0.061
2,027,030	PPE26	*c.241G > A*	*p.Ala81Thr*	0.037	3.019 (0.58-15.721)	0.189
2,027,484	PPE26	*c.695G > A*	*p.Gly232Asp*	0.010	2.375 (0.992–5.688)	0.052
2,041,230	PPE28	*c.1778 A > G*	*p.Tyr593Cys*	0.005	-	-
2,045,310	PE_PGRS32	*c.1533T > C*	*p.Ile511Ile*	0.918	0.556 (0.202–1.531)	0.256
2,168,604	PPE35	*c.2009 C > T*	*p.Pro670Leu*	0.006	**0.202 (0.064–0.633)**	**0.006**
2,226,875	PE_PGRS35	*c.632G > C*	*p.Gly211Ala*	0.018	-	-
2,424,370	PE_PGRS38	*c.469G > A*	*p.Ala157Thr*	0.018	-	-
2,601,760	PE23	*c.1030G > A*	*p.Ala344Thr*	0.792	1.556 (0.203–11.901)	0.670
2,634,732	PPE39	*c.861 C > T*	*p.Ser287Ser*	0.009	2.333 (0.162–33.604)	0.534
2,796,232	PE_PGRS42	*c.1154 C > T*	*p.Ala385Val*	0.006	-	0.999
**Position**	**Gene**	**SNP**	**Effect**	**MAF**	**OR(95%CI)**	**P-value**
2,803,711	PE_PGRS43	*c.2526 C > G*	*p.Gly842Gly*	0.005	-	0.999
2,921,883	PE_PGRS44	*c.333 C > A*	*p.Pro111Pro*	0.007	**12.664 (1.696–94.357)**	**0.013**
2,943,839	PE_PGRS45	*c.1147 A > C*	*p.Ser383Arg*	0.011	0.620 (0.130–2.955)	0.549
2,961,808	PE_PGRS46	*c.634G > A*	*p.Gly212Ser*	0.051	1.254 (0.908–1.731)	0.170
3,054,321	PE_PGRS47	*c.408 A > G*	*p.Gly136Gly*	0.917	**0.197 (0.044–0.893)**	**0.035**
3,163,655	PE_PGRS48	*c.1388G > C*	*p.Gly463Ala*	0.009	1.17 (0/197-6.943)	0.863
3,201,578	PPE45	*c.443 C > T*	*p.Ala148Val*	0.009	0.279 (0.047–1.650)	0.159
3,510,429	PPE52	*c.889 C > G*	*p.Pro297Ala*	0.005	6.052 (0.365-100.387	0.209
3,729,986	PPE54	*c.6950 A > C*	*p.Glu2317Ala*	0.005	-	-
3,742,483	PE_PGRS50	*c.292 C > T*	*p.Pro98Ser*	0.006	0.377 (0.144-1.20)	0.055
3,779,978	PE_PGRS51	*c.1411G > C*	*p.Gly471Arg*	0.006	0.761 (0.134–4.322)	0.758
3,895,585	PPE60	*c.1160 C > T*	*p.Pro387Leu*	0.784	1.264 (0.156–10.218)	0.826
3,929,151	PE_PGRS53	*c.2583 C > T*	*p.Gly861Gly*	0.007	0.624 (0.130–2.992)	0.555
4,032,218	PE_PGRS58	*c.941 C > T*	*p.Ala314Val*	0.760	1.513 (0.851–2.691)	0.159
4,032,625	PE_PGRS58	*c.534 C > A*	*p.Gly178Gly*	0.354	1.163 (0.871–1.553)	0.307
4,032,760	PE_PGRS58	*c.399G > T*	*p.Gly133Gly*	0.022	1.336 (0.395–4.518)	0.641
4,190,456	PPE67	*c.62G > T*	*p.Gly21Val*	0.010	1.96 (0.908–4.231)	0.086
4,351,039	PE35	*c.295G > T*	p.Glu99*	0.944	0.321 (0.068–1.514)	0.151

SNP, single nucleotide polymorphisms; MAF, minor allele frequency; OR, Odds ratio; CI, confidence interval;

-means there is no result in statistical software or the result was too large and nonsense

The 382 strains belonging to lineage 2 formed 77 cross-regional clusters, ranging from 2 to 6 geographic regions. Among the 7 geographic regions, Northern China (31.9%) and Central China (27.3%) exhibited the highest proportion of these cross-regional clusters, followed by and Southwest China (23.1%) and Northwest China (19.5%). In the univariate analysis, 57 SNPs exhibited statistically significant differences between cross-regional and regional clusters (*P* < 0.05). Subsequent multivariate logistic regression analysis identified 10 mutations as influencing factors (*P* < 0.05), with 4 mutation positions recognized as risk factors for cross-regional clusters, including PE_PGRS10 (839,334; *c.884 A > G*; OR, 2.706; 95% CI, 1.081–6.774), PE_PGRS11 (847,613; *c.1455G > C*; OR, 4.342; 95% CI, 1.636–11.525), PE_PGRS47 (3,054,724; *c.811 A > G*; OR, 2.099; 95% CI, 1.211–3.637) and PPE66 (4,189,930; *c.303G > C*; OR, 6.511; 95% CI, (1.679–25.242) (Supplementary Table 3).

**Fig. 2:**
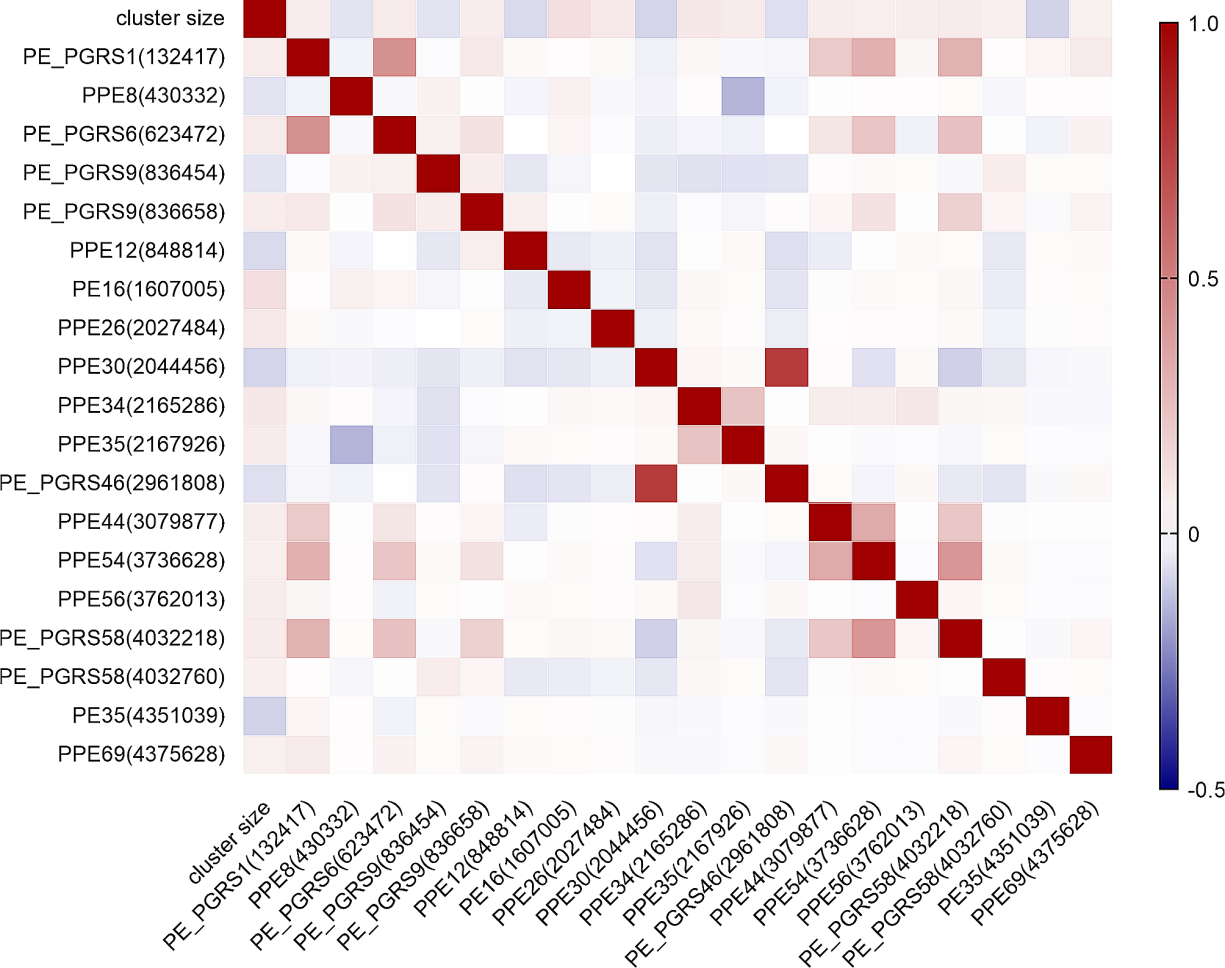
Correlation analysis of PE/PPE gene mutation positions and cluster size

The correlation analysis between mutation sites and cluster size revealed that 19 mutation positions were significantly associated with cluster size (*P* < 0.05), with 13 mutation positions positively correlated with clustering size (*rs* > 0), including PE_PGRS1 (132,417), PE_PGRS6 (623,472), PE_PGRS9 (836,658), PE16 (1,607,005), PPE26 (2,027,484), PPE34 (2,165,286), PPE35 (2,167,926), PPE44 (3,079,877), PPE54 (3,736,628), PPE56 (3,762,013), PE_PGRS58 (4,032,218), PE_PGRS58 (4,032,760) and PPE69 (4,375,628). For further details refer to Fig. 2.

### The effect of PE/PPE gene mutations on transmission of L4 strains

A total of 205 homoplastic SNPs were detected in lineage 4 strains, as presented in Supplementary Table 4 for reference. After excluding homoplastic SNPs with a MAF below 0.005, 74 SNPs in the PE/PPE gene region were selected for in-depth examination. A significant difference in 6 PE/PPE gene mutation positions was detected between clustered and non-clustered strains, as per the single-factor analysis (*P* < 0.05). To further investigate these associations, a multivariate logistic regression analysis was conducted, focusing on the 22 loci with P-values less than 0.2 from the initial univariate analysis. However, no mutations were observed in lineage 4 strains that seemed to facilitate transmission, as displayed in Table 3.

**Table 3:** Analysis of the PE/PPE gene mutations in clustering and non-clustering of lineage 4

Position	Gene	SNP	Effect	MAF	OR (95% CI)	*P*-value
337,820	PE_PGRS4	*c.1254 C > T*	*p.Gly418Gly*	0.085	1.037 (0.565–1.903)	0.906
337,959	PE_PGRS4	*c.1115T > G*	*p.Ile372Ser*	0.045	0.469 (0.175–1.258)	0.133
338,020	PE_PGRS4	*c.1054T > G*	*p.Cys352Gly*	0.059	0.357 (0.122–1.041)	0.059
340,153	PPE3	*c.790G > A*	*p.Glu264Lys*	0.006	2.369 (0.878–6.388	0.088
340,372	PPE3	*c.1009T > C*	*p.Ser337Pro*	0.886	1.048 (0.399–2.750)	0.925
623,508	PE_PGRS6	*c.716 C > G*	*p.Ala239Gly*	0.830	0.749 (0.385–1.461)	0.397
674,416	PE_PGRS7	*c.1501G > A*	*p.Gly501Ser*	0.047	0.949 (0.584–1.544)	0.834
836,426	PE_PGRS9	*c.726 A > C*	*p.Leu242Leu*	0.109	1.014 (0.394–2.613)	0.976
836,538	PE_PGRS9	*c.838 A > G*	*p.Asn280Asp*	0.508	0.982 (0.510–1.890)	0.957
836,658	PE_PGRS9	*c.958 A > G*	*p.Thr320Ala*	0.769	0.943 (0.438–2.030)	0.881
837,033	PE_PGRS9	*c.1333 A > G*	*p.Thr445Ala*	0.910	0.702 (0.172–2.865)	0.622
838,990	PE_PGRS10	*c.540 C > G*	*p.Ala180Ala*	0.288	1.027 (0.329–3.209)	0.963
839,334	PE_PGRS10	*c.884 A > G*	*p.Lys295Arg*	0.369	0.465 (0.099–2.187)	0.332
839,348	PE_PGRS10	*c.898 A > G*	*p.Ser300Gly*	0.348	2.013 (0.431–9.394)	0.374
927,110	PE_PGRS13	*c.1750 A > G*	*p.Ser584Gly*	0.297	1.839 (0.642–5.268)	0.256
927,385	PE_PGRS13	*c.2025 A > G*	*p.Gly675Gly*	0.330	1.791 (0.521 6.162)	0.355
2,026,619	PE18	*c.143 C > G*	*p.Ser48Trp*	0.010	1.865 (0.826–4.211)	0.134
2,165,286	PPE34	*c.2026T > G*	*p.Ser676Ala*	0.902	1.891 (0.599–5.966)	0.277
2,387,733	PE_PGRS37	*c.240 A > G*	*p.Glu80Glu*	0.893	0.853 (0.361–2.020)	0.718
3,054,724	PE_PGRS47	*c.811 A > G*	*p.Ser271Gly*	0.244	0.779 (0.294–2.069)	0.617
3,736,628	PPE54	*c.308 A > C*	*p.Glu103Ala*	0.927	0.169 (0.016–1.785)	0.139
3,940,802	PE_PGRS55	*c.1186 A > G*	*p.Asn396Asp*	0.296	1.493 (0.668–3.336)	0.328

SNP, single nucleotide polymorphisms; MAF, minor allele frequency; OR, Odds ratio; CI, confidence interval;

Furthermore, 25 lineage 4 strains grouped into 9 cross-regional clusters, with strains in each cluster spanning two different geographic regions. After conducting univariate analysis, 9 mutation sites were selected for a multivariate regression analysis, revealing that 4 positions were significantly associated with cross-regional clusters (*P* < 0.05). PE_PGRS4 (338,100; *c.974 A > G*; OR, 6.090; 95% CI, 1.702–21.793) and PPE13 (976,897; *c.1307 A > C*; OR, 3.505; 95% CI,1.103–11.132) considered as risk factors for cross-regional transmission of strains (Supplementary Table 5). Due to the low prevalence of lineage 4 strains in China and the relatively small sample size, we did not further analyze the transmission cluster size of lineage 4 strains.

## Discussion

We analyzed 3202 domestic isolates (including the 1447 Shandong isolates and 1755 publicly available isolates) in this study. The genomic analysis of *M. tuberculosis* across the country reveals that lineage 2 is the predominant strain, contributing significantly to the tuberculosis burden in China, followed by lineage 4. Moreover, lineage 2 strains had a considerably larger proportion of strains in transmission clusters than lineage 4 strains. In subsequent analyses, we identified 4 PE/PPE gene mutations associated with the spread of lineage 2 strains, but no mutations were found to enhance the transmission of lineage 4 strains.

Homoplastic mutations are mutations independently occurring in different clades of an organism. Homoplastic changes may be a result of convergence evolution due to selective pressures [39]. Previous reports have shown that homoplastic SNPs were present in all functional categories of genes, with PE/PPE gene family having the highest ratio of homoplastic SNPs compared to the total SNPs identified in the same functional category [34], but the relationship between these SNPs within the PE/PPE gene region and strain transmission has not been described. In our study, the results of homoplasy analysis of strains showed that 1,141 homoplastic SNPs were identified in strains of lineage 2 and 78 homoplastic SNPs were confirmed in strains of lineage 4, respectively.

The PE/PPE genes are especially abundant in pathogenic mycobacteria, suggesting that they play a major role in mycobacterial survival and pathogenesis, although the precise function of these proteins is largely unknown [6, 40]. Based on our findings, we observed a missense mutation (*c.46G > A*, *p.Ala16Thr*) at position 190,394 of PE4.

(Rv0160c), and another missense mutation (*c.620T > G*, *p.Leu207Arg*) at position 1,607,005 of PE16 (Rv1430), which have been associated with increased risk of transmission of *M. tuberculosis* within lineage 2. Despite the lingering uncertainties surrounding the exact function of PE4, recent study has shed light on its role in enhancing mycobacterial survival within macrophages [41]. PE16, a member of the serine hydrolase superfamily with esterase activity, is particularly notable for its ability to hydrolyze short- to medium-chain fatty acid esters [42]. Both PE4 and PE16 play pivotal roles in the progression of mycobacterial infections. To fully comprehend the mechanisms through which these mutations facilitate transmission, further in-depth research is imperative. A synonymous mutation at position 839,194 of PE_PGRS10 (Rv0747) (*c.744 A > G*, *p.Thr248Thr*) has been found to affect the transmission of lineage 2 isolates. Mazandu and Mulder [43] have predicted that PE_PGRS10 is involved in lipid metabolism, although this has yet to be experimentally verified, an important feature of mycobacterial pathogenicity. This synonym mutation can affect the spread of *M. tuberculosis*, indicating that the synonym mutations of PE/PPE genes are not all neutral mutations, which is consistent with the previous research results that the synonym mutations of yeast genes are mostly strong non-neutral mutations [44].

The protein functions of PE_PGRS are unique to mycobacteria and are secreted or cell surface associated [45]. This suggests that they could be involved in mediating the interaction between the macrophages and the bacteria [46–48]. PE_PGRS proteins were translocated through the plasmatic membrane in an ESX5-dependent mechanism [49, 50], once in the outer membrane PE_PGRS proteins may “float” on the micromembrane outer leaflet and may possibly be released to exert their activity. The cell wall-anchored/secretory protein PE_PGRS 11 (Rv0754) exhibits a significant interaction with TLR2, driving the maturation and activation of human dendritic cells (DCs), thereby enhancing their capacity to stimulate CD4 + T cells [20]. Analysis of the effects of deletion or over-expression of PE_PGRS47 (Rv2741) implicated this protein in the inhibition of autophagy in infected host phagocytes. As a functionally significant and non-redundant bacterial component, PE_PGRS47 contributes to the modulation of both innate and adaptive immune responses. This finding implicates PE_PGRS47 as a potential target for enhancing antigen presentation and fostering protective immunity during vaccination or infection [51]. A missense mutation (*c.1455G > C, p.Glu485Asp*) at position 847,613 of PE_PGRS11, and another missense mutation (*c.811 A > G, p.Ser271Gly*) at position 3,054,724 of PE_PGRS47 have been found to promote the cross-regional spread of lineage 2 isolates.

A previous study of the influence of genomic variants on *M. tuberculosis* transmission in Malawi showed significant convergent evolution in a mutation in PPE54, which associated with transmission [24]. Notably, 68.7% of the strains in this study belonged to lineage 4, and only 3.8% belonged to lineage 2. However, in our study, we did not find that the mutation of PE54 could promote the spread of lineage 4 strains in China. One possible explanation for this could be the bacteriological diversity of the lineage 4 sublineages, which show complex population structure with 21 sublineages and 15 internal groups [35, 52]. Another possible reason for this difference is the limited sample size of lineage 4, which might have been inadequate to identify less common lineage 4 strains or associated mutations. A larger dataset might potentially yield more precise findings, suggesting that the current sample size might have introduced a degree of inaccuracy.

Our study is the first to explore the association of PE/PPE gene mutations with *M. tuberculosis* transmission in China. Nevertheless, we could illustrate some limitations. The distribution of *M. tuberculosis* isolates in the current dataset may not accurately reflect the prevalence of TB in some areas, but our data have a large sample size and broad geographical distribution which allowed us to analyze how *M. tuberculosis* spread across the whole country. Additionally, due to the low prevalence of lineage 4 in China and the relatively small sample size, we did not subdivide the transmission cluster sizes of lineage 4 strains. The higher sensitivity of lineage specific analysis may require a larger sample size to be achieved.

## Conclusion

In summary, our work highlights two main *M. tuberculosis* lineages of transmission in China, as well as some PE/PPE gene mutations can increase the risk of MTB transmission in lineages 2 and 4, respectively, providing valuable insights for the treatment of TB. We believe that the PE/PPE family will remain a highly active area of research with various exciting features yet to be discovered.

### Electronic supplementary material

Below is the link to the electronic supplementary material.

Supplementary Material 1

Supplementary Material 2

Supplementary Material 3

Supplementary Material 4

Supplementary Material 5

Supplementary Material 6

## Data Availability

The newly sequenced whole genome dataset of 1,447 *M. tuberculosis* strains was deposited in the NCBI Bio Project (accession number is PRJNA1002108), and 1755 other isolates were downloaded from the European Nucleotide Archive repository. (accession numbers are provided in Supplementary Table 1). Any additional data are available from the corresponding authors upon reasonable request.
