# Supplementary material for: PE/PPE mutations in the transmission of Mycobacterium tuberculosis in China revealed by whole genome sequencing
Source: BMC Microbiol. 2024 Jun 10;24:206. doi: 10.1186/s12866-024-03352-y (PMC11163795; doi:10.1186/s12866-024-03352-y)
Supplement: Supplementary file 3 — Supplementary Material 3 [file 12866_2024_3352_MOESM3_ESM.doc]

**Supplementary table 3.** Analysis of the PE/PPE gene mutations within cross-regional and regional clusters of lineage 2.

| **Position** | **Gene** | **SNP** | **Effect** | **MAF** | **OR(95%CI)** | **P-value** |
| --- | --- | --- | --- | --- | --- | --- |
| 132417 | PE_PGRS1 | c.1036C>G | p.Arg346Gly | 0.895 | 1.149 (0.453-2.915) | 0.769 |
| 188800 | PE3 | c.40A>G | p.Thr14Ala | 0.928 | 0.489 (0.064-3.741) | 0.491 |
| 190394 | PE4 | c.46G>A | p.Ala16Thr | 0.009 | 0.355 (0.099-1.277) | 0.113 |
| 333892 | PE_PGRS3 | c.2419C>G | p.Arg807Gly | 0.375 | 1.044 (0.594-1.832) | 0.882 |
| 334641 | PE_PGRS3 | c.1670C>G | p.Ala557Gly | 0.054 | 0.727 (0.305-1.73) | 0.471 |
| 340372 | PPE3 | c.1009T>C | p.Ser337Pro | 0.886 | 1.633 (0.655-4.072) | 0.293 |
| 374353 | PPE6 | c.1359T>C | p.Arg453Arg | 0.641 | 0.655 (0.235-1.829) | 0.419 |
| 430332 | PPE8 | c.4348T>C | p.Phe1450Leu | 0.013 | - | - |
| 623472 | PE_PGRS6 | c.680A>G | p.Asp227Gly | 0.807 | **0.477 (0.232-0.98)** | **0.044** |
| 623508 | PE_PGRS6 | c.716C>G | p.Ala239Gly | 0.830 | 0.538 (0.283-1.022) | 0.058 |
| 673564 | PE_PGRS7 | c.2353G>A | p.Ala785Thr | 0.190 | 0.724 (0.452-1.158) | 0.177 |
| 836272 | PE_PGRS9 | c.572A>G | p.Glu191Gly | 0.386 | 1.405 (0.679-2.908) | 0.360 |
| 836291 | PE_PGRS9 | c.591A>G | p.Gly197Gly | 0.369 | 0.823 (0.408-1.658) | 0.585 |
| 836426 | PE_PGRS9 | c.726A>C | p.Leu242Leu | 0.109 | 0.792 (0.271-2.315) | 0.670 |
| 836454 | PE_PGRS9 | c.754A>G | p.Thr252Ala | 0.111 | 2.238 (0.729-6.872) | 0.159 |
| 836538 | PE_PGRS9 | c.838A>G | p.Asn280Asp | 0.508 | 1.430 (0.925-2.212) | 0.108 |
| 836658 | PE_PGRS9 | c.958A>G | p.Thr320Ala | 0.769 | 1.438 (0.876-2.363) | 0.151 |
| 838990 | PE_PGRS10 | c.540C>G | p.Ala180Ala | 0.288 | 0.892 (0.486-1.639) | 0.713 |
| 839194 | PE_PGRS10 | c.744A>G | p.Thr248Thr | 0.302 | 0.889 (0.514-1.539) | 0.675 |
| 839334 | PE_PGRS10 | c.884A>G | p.Lys295Arg | 0.369 | **2.706 (1.081-6.774)** | **0.033** |
| 839348 | PE_PGRS10 | c.898A>G | p.Ser300Gly | 0.348 | 0.382 (0.146-1.003) | 0.051 |
| 839684 | PE_PGRS10 | c.1234G>A | p.Ala412Thr | 0.012 | - | - |
| 839982 | PE_PGRS10 | c.1532C>G | p.Ala511Gly | 0.006 | 1.337 (0.286-6.248) | 0.712 |
| 847613 | PE_PGRS11 | c.1455G>C | p.Glu485Asp | 0.017 | **4.342 (1.636-11.525)** | **0.003** |
| 925453 | PE_PGRS13 | c.93C>T | p.Ala31Ala | 0.387 | 1.123 (0.658-1.916) | 0.670 |
| 927110 | PE_PGRS13 | c.1750A>G | p.Ser584Gly | 0.297 | 1.119 (0.606-2.069) | 0.719 |
| 927385 | PE_PGRS13 | c.2025A>G | p.Gly675Gly | 0.330 | 0.949 (0.469-1.919) | 0.884 |
| 969762 | PE_PGRS15 | c.483G>C | p.Gly161Gly | 0.716 | **0.335 (0.171-0.656)** | **0.001** |
| 1020942 | PPE14 | c.388A>C | p.Ser130Arg | 0.020 | 0.571 (0.12-2.718) | 0.482 |
| 1212432 | PE_PGRS21 | c.873C>A | p.Gly291Gly | 0.307 | 0.993 (0.563-1.752) | 0.981 |
| 1217157 | PE_PGRS22 | c.689A>C | p.Asp230Ala | 0.383 | 1.273 (0.76-2.132) | 0.359 |
| 1218896 | PE_PGRS22 | c.2428G>A | p.Gly810Ser | 0.399 | 0.823 (0.472-1.433) | 0.491 |
| 1561939 | PPE20 | c.171G>C | p.Glu57Asp | 0.743 | - | 0.999 |
| 1607005 | PE16 | c.620T>G | p.Leu207Arg | 0.020 | **0.070 (0.011-0.452)** | **0.005** |
| 1618978 | PE_PGRS26 | c.707A>G | p.Asp236Gly | 0.354 | 0.846 (0.45-1.591) | 0.604 |
| 1655943 | PE_PGRS29 | c.779T>A | p.Phe260Tyr | 0.025 | - | 0.997 |
| 1862424 | PE_PGRS30 | c.2959G>A | p.Gly987Ser | 0.006 | - | 0.999 |
| 2027030 | PPE26 | c.241G>A | p.Ala81Thr | 0.037 | - | 0.999 |
| 2027484 | PPE26 | c.695G>A | p.Gly232Asp | 0.010 | 1.237 (0.337-4.547) | 0.749 |
| 2044456 | PPE30 | c.1073T>G | p.Leu358Arg | 0.044 | 0.762 (0.241-2.414) | 0.644 |
| **Position** | **Gene** | **SNP** | **Effect** | **MAF** | **OR(95%CI)** | **P-value** |
| 2061958 | PE_PGRS33 | c.717T>C | p.Ala239Ala | 0.286 | 1.363 (0.716-2.592) | 0.346 |
| 2167926 | PPE35 | c.2687T>C | p.Leu896Ser | 0.791 | 9.477 (0.935-96.046) | 0.057 |
| 2387733 | PE_PGRS37 | c.240A>G | p.Glu80Glu | 0.893 | 0.821 (0.353-1.907) | 0.646 |
| 2634732 | PPE39 | c.861C>T | p.Ser287Ser | 0.009 | - | 0.999 |
| 2802041 | PE_PGRS43 | c.4196G>A | p.Gly1399Asp | 0.516 | 1.145 (0.692-1.894) | 0.598 |
| 2835984 | PE26 | c.200T>C | p.Val67Ala | 0.626 | 1.380 (0.466-4.085) | 0.561 |
| 2921883 | PE_PGRS44 | c.333C>A | p.Pro111Pro | 0.007 | - | 0.999 |
| 2943839 | PE_PGRS45 | c.1147A>C | p.Ser383Arg | 0.011 | 14.412 (0.3-693.121) | 0.177 |
| 2961808 | PE_PGRS46 | c.634G>A | p.Gly212Ser | 0.051 | **0.362 (0.15-0.876)** | **0.024** |
| 3054321 | PE_PGRS47 | c.408A>G | p.Gly136Gly | 0.917 | 1.924 (0.446-8.311) | 0.380 |
| 3054724 | PE_PGRS47 | c.811A>G | p.Ser271Gly | 0.244 | **2.099 (1.211-3.637)** | **0.008** |
| 3162805 | PE_PGRS48 | c.538C>G | p.Arg180Gly | 0.583 | 1.723 (0.991-2.996) | 0.054 |
| 3163655 | PE_PGRS48 | c.1388G>C | p.Gly463Ala | 0.009 | 3.336 (0.063-175.869) | 0.552 |
| 3201578 | PPE45 | c.443C>T | p.Ala148Val | 0.009 | - | 0.999 |
| 3735540 | PPE54 | c.1396A>G | p.Ile466Val | 0.143 | **0.684 (0.469-0.999)** | **0.049** |
| 3736628 | PPE54 | c.308A>C | p.Glu103Ala | 0.927 | 0.592 (0.15-2.345) | 0.456 |
| 3737735 | PE_PGRS49 | c.266C>T | p.Pro89Leu | 0.014 | - | 0.998 |
| 3741572 | PE_PGRS50 | c.1203C>T | p.Gly401Gly | 0.560 | 1.141 (0.614-2.03) | 0.654 |
| 3895585 | PPE60 | c.1160C>T | p.Pro387Leu | 0.784 | 0.549 (0.053-5.638) | 0.613 |
| 3927595 | PE_PGRS53 | c.1027G>A | p.Ala343Thr | 0.108 | 1.205 (0.78-1.861) | 0.401 |
| 3928281 | PE_PGRS53 | c.1713T>C | p.Gly571Gly | 0.005 | 2.297 (0.333-15.867) | 0.399 |
| 3940802 | PE_PGRS55 | c.1186A>G | p.Asn396Asp | 0.296 | 0.567 (0.319-1.011) | 0.054 |
| 3941103 | PE_PGRS55 | c.1487G>A | p.Gly496Asp | 0.022 | 1.721 (0.277-10.684) | 0.560 |
| 3944217 | PE_PGRS56 | c.406G>A | p.Ala136Thr | 0.018 | 0.293 (0.054-1.58) | 0.153 |
| 4032625 | PE_PGRS58 | c.534C>A | p.Gly178Gly | 0.354 | 0.971 (0.561-1.68) | 0.915 |
| 4032760 | PE_PGRS58 | c.399G>T | p.Gly133Gly | 0.022 | 0.658 (0.056-7.759) | 0.740 |
| 4189930 | PPE66 | c.303G>C | p.Leu101Phe | 0.007 | **6.511 (1.679-25.242)** | **0.007** |
| 4351039 | PE35 | c.295G>T | p.Glu99* | 0.944 | **0.016 (0.002-0.15)** | **<0.001** |

SNP, single nucleotide polymorphisms; MAF, minor allele frequency; OR, Odds ratio; CI, confidence interval;

-means there is no result in statistical software or the result was too large and nonsense.
