# Supplementary material for: PE/PPE mutations in the transmission of Mycobacterium tuberculosis in China revealed by whole genome sequencing
Source: BMC Microbiol. 2024 Jun 10;24:206. doi: 10.1186/s12866-024-03352-y (PMC11163795; doi:10.1186/s12866-024-03352-y)
Supplement: Supplementary file 5 — Supplementary Material 5 [file 12866_2024_3352_MOESM5_ESM.doc]

**Supplementary table 5.** Analysis of the PE/PPE gene mutations within cross-regional and regional clusters of lineage 4.

| **Position** | **Gene** | **SNP** | **Effect** | **MAF** | **OR(95%CI)** | **P-value** |
| --- | --- | --- | --- | --- | --- | --- |
| 187439 | PE3 | *c.1401C>G* | *p.Ile467Met* | 0.057 | 0.828 (0.211-3.255) | 0.787 |
| 338100 | PE_PGRS4 | *c.974A>G* | *p.Asn325Ser* | 0.072 | **6.09 (1.702-21.793)** | **0.005** |
| 340372 | PPE3 | *c.1009T>C* | *p.Ser337Pro* | 0.886 | 1.072 (0.148-7.744) | 0.945 |
| 624025 | PE_PGRS6 | *c.1233C>G* | *p.Ser411Arg* | 0.009 | 1.812 (0.245-13.423) | 0.561 |
| 839123 | PE_PGRS10 | *c.673A>G* | *p.Arg225Gly* | 0.219 | 0.477 (0.105-2.165) | 0.337 |
| 928300 | PE_PGRS14 | *c.2186G>A* | *p.Gly729Asp* | 0.019 | **0.095 (0.009-0.986)** | **0.049** |
| 976897 | PPE13 | *c.1307A>C* | *p.Gln436Pro* | 0.027 | **3.505 (1.103-11.132)** | **0.033** |
| 3162805 | PE_PGRS48 | *c.538C>G* | *p.Arg180Gly* | 0.583 | **0.133 (0.022-0.799)** | **0.027** |
| 3736628 | PPE54 | *c.308A>C* | *p.Glu103Ala* | 0.927 | 0.168 (0.011-2.535) | 0.198 |

SNP, single nucleotide polymorphisms; MAF, minor allele frequency; OR, Odds ratio; CI, confidence interval;

-means there is no result in statistical software or the result was too large and nonsense.
