# Supplementary material for: PE/PPE mutations in the transmission of Mycobacterium tuberculosis in China revealed by whole genome sequencing
Source: BMC Microbiol. 2024 Jun 10;24:206. doi: 10.1186/s12866-024-03352-y (PMC11163795; doi:10.1186/s12866-024-03352-y)
Supplement: Supplementary file 6 — Supplementary Material 6 [file 12866_2024_3352_MOESM6_ESM.doc]

**Supplementary table legends**

**Supplementary table 1**. Sequence data and lineage assignments according to Tbprofile for 3202 Chinese isolates.

**Supplementary table 2.** Homoplastic SNPs detected in the PE/PPE gene regions of lineage 2 strains.

**Supplementary table 3**. Analysis of the PE/PPE gene mutations within cross-regional and regional clusters of lineage 2.

**Supplementary table 4.** Homoplastic SNPs detected in the PE/PPE gene regions of lineage 4 strains.

**Supplementary table 5**. Analysis of the PE/PPE gene mutations within cross-regional and regional clusters of lineage 4.
